# Nanomaterial assisted natural killer cell therapy

**DOI:** 10.3389/fimmu.2025.1558701

**Published:** 2025-05-05

**Authors:** Xuan Yu, Xiaobo Chen, Yanlong Yang, Yingsong Tian, Jie Jia, Xinghe Tong

**Affiliations:** ^1^ Department of Thoracic Surgery, The First Affiliated Hospital of Kunming Medical University, Kunming, Yunnan, China; ^2^ Clinical Medical Research Center, The First Affiliated Hospital of Kunming Medical University, Kunming, Yunnan, China

**Keywords:** natural killer cells, NK cell therapy, CAR-NK, nanometer material, tumor immunotherapy

## Abstract

The rising incidence of cancer has heightened interest in immune cell therapy, particularly the role of natural killer (NK) cells, which are essential components of the immune system. Their applications in tumor treatment have expanded significantly, especially with the incorporation of nanomaterials. This review comprehensively examines NK cell biology, encompassing aspects such as classification, distribution, receptor activation, and mechanisms of cytotoxicity. It also explores various NK cell therapies, including their sources, methods of acquisition, expansion techniques, Chimeric antigen receptor-Natural Killer cell (CAR-NK) technology, gene editing strategies, and combination therapies. Additionally, the review discusses the utilization of nanomaterials in NK cell therapy, focusing on nanoparticle-assisted immune regulation and the modulation of the tumor microenvironment. While NK cell therapy holds promise, CAR-NK technology presents certain limitations. The integration of nanomaterials offers potential strategies to enhance therapeutic efficacy. Future research should prioritize the optimization of NK cell therapy, address the limitations associated with CAR-NK technology, investigate the mechanisms of nanomaterials, and develop more effective nanomaterials to improve clinical outcomes.

## 1 Introduction

With the rising incidence of cancer-related deaths and newly diagnosed cases, there is renewed focus on immune cells. Natural killer (NK) cells, classified as Innate Lymphoid Cells (ILCs) (1), play a pivotal role in the human immune system (2).They exhibit potent anti-tumor and antiviral effects and also perform additional functions through the production of chemokines (CCL3/CCL4) and cytokines (Interferon γ, tumor necrosis factor α) (3). Moreover, NK cells are actively involved in hypersensitivity reactions and autoimmune diseases (4, 5).

Following promising results in hematological malignancies, there has been increased interest in employing NK cell therapy for the treatment of solid tumors. In recent years, research on NK cell therapy for solid tumors has gradually expanded. Additionally, the integration of nanomaterials into this research area has emerged as a prominent focus, with numerous strategies exploring the combination of NK cell therapy and nanomaterials. Bioactive nanomaterials, which exhibit good biocompatibility and degradability, can address the drawbacks of CAR-NK cells in tumor treatment. Nanoparticles in this therapy assist in immune regulation, act as carriers for enhanced efficacy, and improve NK cell homing. They also play a role in regulating the tumor microenvironment. While extracellular vesicle-related nanomaterials demonstrate anti-tumor effects, challenges remain in their production and delivery. Plant-derived nanomaterials are emerging as a future focus. Furthermore, nanoparticle-modified NK cells and nanomaterial-coated cell membranes can enhance therapy, with different modifications serving distinct functions.

Here we discuss NK cell therapy and its mechanisms as well as the role of related nanomaterials.

## 2 NK cell biology

### 2.1 Classification and distribution

Human natural killer (NK) cells are primarily distributed in peripheral blood, liver, spleen, lungs, and lymph nodes (6). Initially, it was believed that NK cells matured solely in the bone marrow. However, advancing research has provided increasing evidence that NK cells can also mature in secondary lymphoid tissues (SLT), such as tonsils and lymph nodes (7). It is important to note that NK cells exhibiting distinct phenotypes display different distribution patterns. CD56dimCD16+ NK cells are more abundant and predominant in peripheral blood; however, they do not truly reside within tissues (8). In contrast, CD56brightCD16− NK cells are more prevalent and widely distributed in both human tissues and lymphoid tissues. Furthermore, functional disparities exist between these two subsets: CD56dimCD16+ NK cells demonstrate high cytotoxic activity even at rest and can produce cytokines while in a quiescent state (9), whereas CD56brightCD16− NK cells require monocyte activation, specifically through the combined action of cytokines such as IL-2, IL-12, IL-15, and IL-18, to acquire cytotoxic activity and produce cytokines (10). Notably, CD56brightCD16− NK cells constitute the primary cell population in inflammatory and cancerous tissues (11).

### 2.2 Receptors and activation

**Figure 1:**
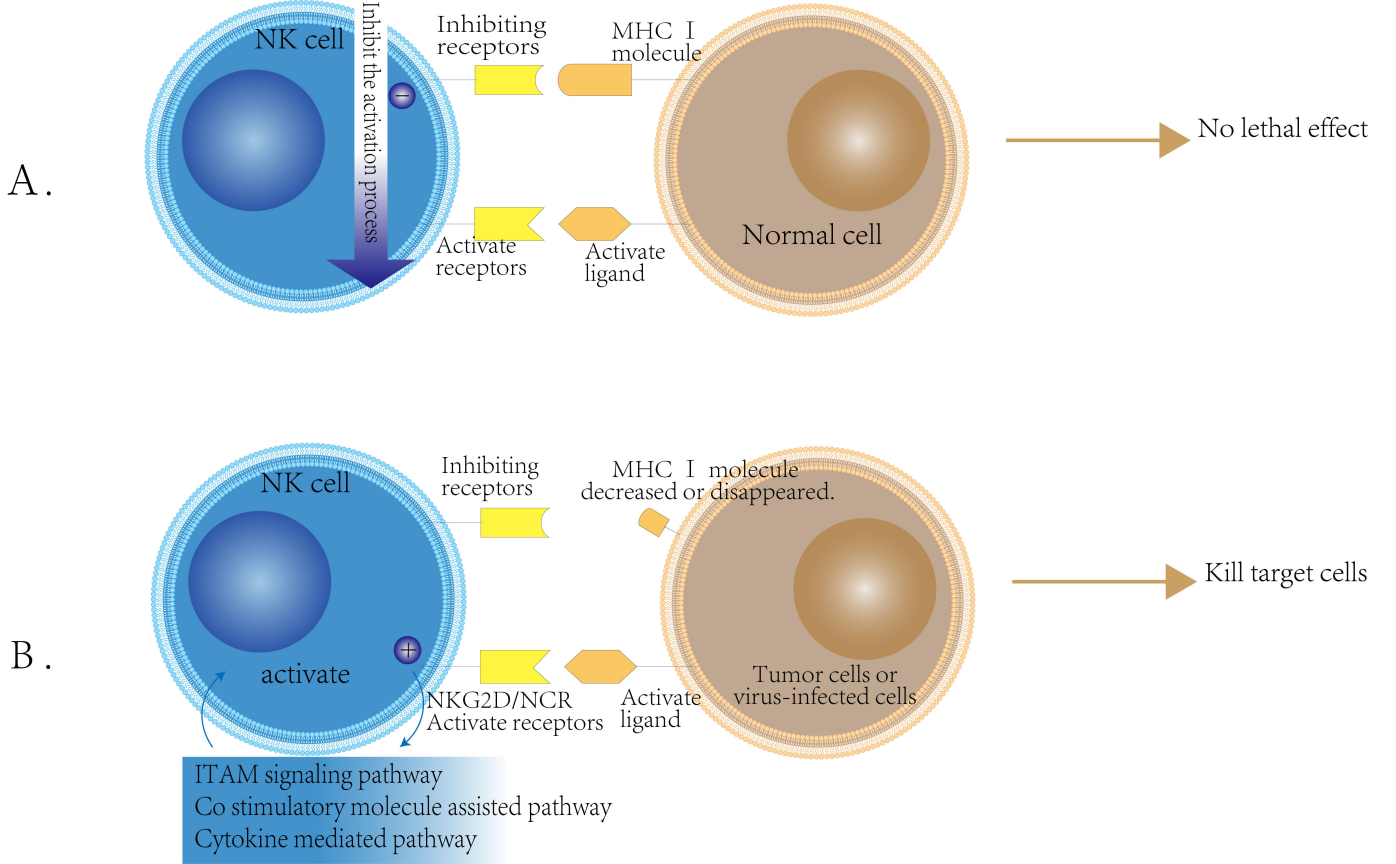
NK cell activity regulation mechanism schematic. **(A)** Inhibition Mechanism of NK Cells: NK cells possess inhibitory receptors for MHC class I molecules on their surface. When these receptors bind to the normal cell surface MHC class I molecules, they transmit inhibitory signals that prevent NK cell activation. As a result, NK cells do not exert cytotoxic effects on normal cells. **(B)** Activation Mechanism of NK Cells: The activation of NK cells occurs when the expression of MHC class I molecules on the surface of tumor cells or virus-infected cells is reduced or absent. In this scenario, the inhibitory receptors on NK cells are not engaged, while the expression of ligands for activating receptors, such as NKG2D and NCR, is upregulated on the surface of these target cells. This leads to the activation of NK cells, which then kill target cells through ITAM signaling, costimulatory molecular assistance, and cytokine-mediated pathways.

Natural killer (NK) cells primarily function to eliminate cells that exhibit reduced or absent expression of major histocompatibility complex class I (MHC I) molecules (12). The activation of NK cells is predominantly regulated by a balance between activating and inhibitory receptors (13). Under normal physiological conditions, self-tissue cells typically express MHC class I molecules. In this context, the inhibitory receptors on the surface of NK cells are predominant, thereby suppressing the activity of activating receptors and preventing NK cells from targeting their own healthy tissue (14). However, in the event of viral infection or cancerous transformation, MHC class I molecules may be lost or downregulated (15). (**Figure 1**).Concurrently, the expression of other non-MHC class I molecules recognized by NK cells may become aberrant or upregulated (16). This alteration can result in the inactivation of NK cell inhibitory receptors, leading to the activation of their activating receptors and, consequently, the activation of NK cells, which enables them to target and kill affected cells (17). This phenomenon is referred to as the ‘missing self’ mechanism (18).

### 2.3 The killing function of NK cells against target cells

**Figure 2:**
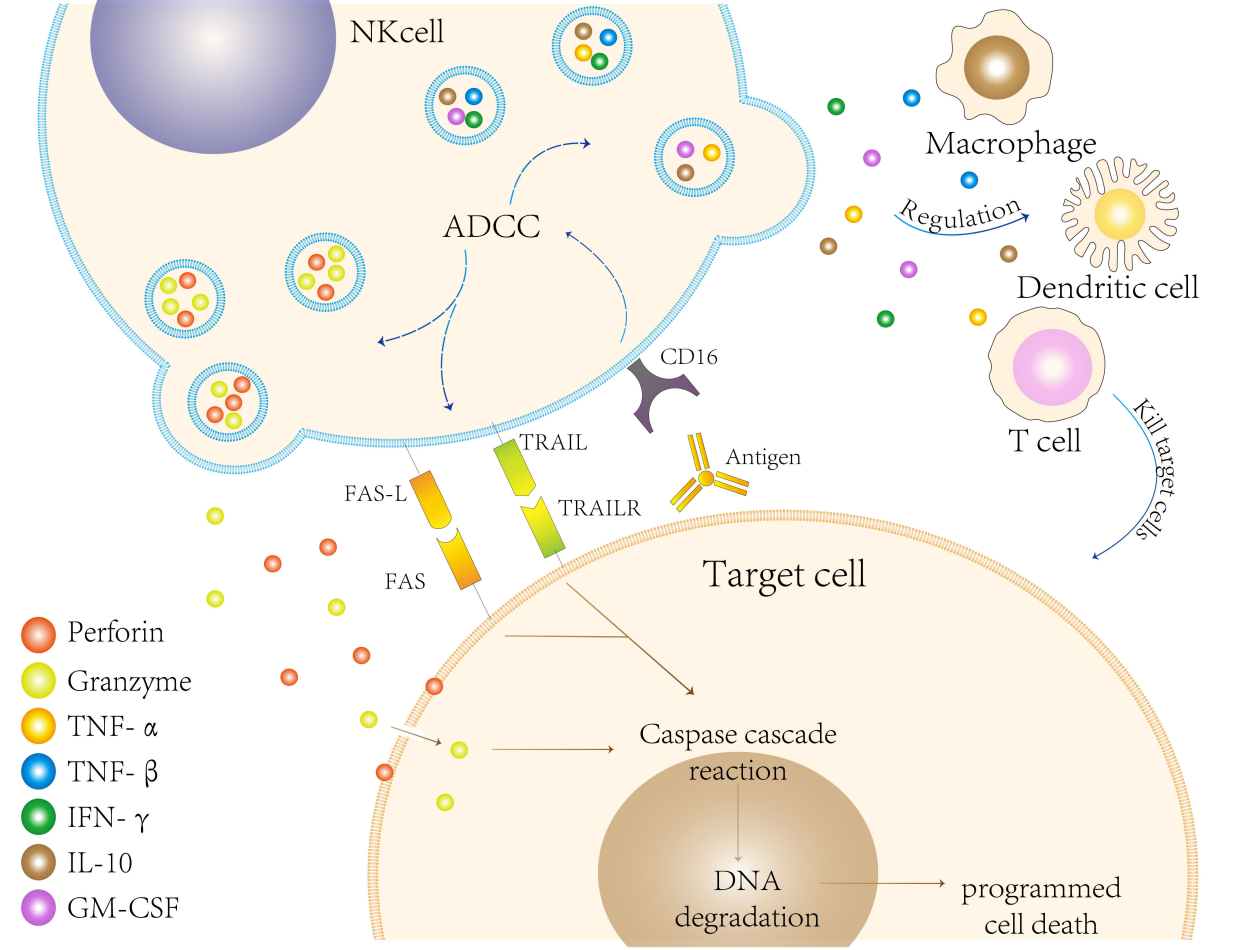
Natural killer (NK) cells eliminate target cells through various mechanisms. First, they can engage in antibody-dependent cellular cytotoxicity (ADCC) by recognizing and binding to the Fc segment of antibodies via CD16 present on their surfaces. Second, NK cells release perforin and granzymes, which induce programmed cell death in target cells. This process activates the caspase cascade, ultimately leading to DNA degradation. Third, FAS ligand (FAS-L) on the surface of NK cells binds to FAS on target cells, while the release of TRAIL binds to TRAIL receptors (TRAILR) on target cells, both of which can induce apoptosis. Collectively, these mechanisms illustrate the effectiveness and diversity of NK cells in targeting and eliminating cells during immune defense. Additionally, NK cells further modulate the immune response by influencing other immune cells, such as macrophages and dendritic cells, as well as regulating cytokine production.

To evade the cytotoxic activity of CD8+ T cells, infected or cancerous cells downregulate the expression of MHC class I molecules (19). NK cells lyse target cells by two primary mechanisms (20): Firstly, they utilize perforin, a membrane-disrupting protein, and granzyme, a proteolytic enzyme, for specific lysis target cells. Upon recognizing the target cells, NK cells become activated, leading to the degranulation and release of perforin and granzyme through the immune synapse between them (21). Perforin creates pores in the cell membrane of the target cells, granzyme to enter and induce apoptosis (22); Secondly, NK cells can also induce death in target cells by modulating cytokines such as tumor necrosis factors, FAS- FASLG (FAS ligand), and TNF-TRAIL (TNF-related apoptosis-inducing ligand). Activated NK cells express corresponding ligands that bind to death receptors on the surfaces of target cells, triggering caspase-mediated enzymatic cascade reactions that result in apoptosis (23) (**Figure 2**).

## 3 NK cell therapy

### 3.1 Origin of NK cells

In the realm of NK cell therapy research, the source of NK cells is of paramount importance, encompassing peripheral blood, bone marrow, umbilical cord blood, and induced pluripotent stem cells (iPSCs).

Peripheral blood NK cells are frequently utilized in cell therapy due to their ease of collection and capacity for expansion (24). These NK cells primarily originate from the bone marrow and exhibit high cytotoxicity along with rapid responsiveness. Through *in vitro* culture and stimulation with cytokines such as IL-2 and IL-15, NK cells can be expanded on a large scale for clinical applications (25). Bone marrow serves as the primary site for NK cell production. NK cells derived from umbilical cord blood share similar characteristics with those derived from bone marrow stromal cells or hematopoietic stem cells (26), and they have the capability to induce mature NK cells *in vitro* (27). Furthermore, NK cells from this source demonstrate robust proliferation and cytotoxic activity, making them suitable candidates for cell therapy (28). iPSCs, with their capacity for unlimited proliferation, can differentiate into various cell lineages, including NK cells, *in vitro* (29). Advances in gene editing technology hold promise for enhancing the anti-tumor activity of NK cells derived from iPSCs (30). These NK cells not only provide an abundant source but also present opportunities to improve therapeutic efficacy against specific tumors through genetic modifications (31).

In summary, the source of NK cells significantly influences their functionality and therapeutic effectiveness. The success of NK cell therapy relies on the judicious selection of the appropriate NK cell source and the optimization of their amplification and activation conditions (32). Future research is anticipated to explore the characteristics of NK cells from diverse sources and their potential applications in tumor immunotherapy.

### 3.2 Acquisition and expansion of NK cells

The expansion of natural killer (NK) cells is pivotal in clinical treatment, particularly within the realm of cancer immunotherapy. An effective strategy for NK cell expansion not only increases the overall cell count but also enhances their functional capabilities and specificity, thereby improving the efficacy of immunotherapeutic interventions (33). Current methodologies for NK cell expansion encompass cytokine stimulation, co-culture techniques, and the application of small molecule compounds.

Cytokine stimulation strategies for NK cell expansion typically emphasize interleukin-2 (IL-2) and interleukin-15 (IL-15) as traditional options. IL-2 is widely recognized for its capacity to promote the proliferation and activation of NK cells (34). Furthermore, IL-15 not only improves the survival and functional capacity of NK cells (35) but also significantly enhances their anti-tumor activity (36). Co-culturing NK cells with tumor cells or other immune cells (37), such as dendritic cells, can bolster the proliferation and anti-tumor activity of NK cells through beneficial cell-cell interactions (38). Additionally, certain small molecule compounds, including histone deacetylase (HDAC)inhibitors and transforming growth factor-beta (TGF-β) inhibitors, can also augment the function and proliferation of NK cells by modulating cell signaling pathways (39, 40).

### 3.3 CAR-NK

#### 3.3.1 What is CAR-NK

Chimeric Antigen Receptor Natural Killer (CAR-NK) cell therapy involves the genetic modification of natural killer (NK) cells using chimeric antigen receptors. This modification enhances the tumor specificity of NK cells and extends their duration of action within the tumor microenvironment (41). Furthermore, CAR-NK engineering can optimize lymphocyte activation signals and utilize specific intracellular signaling molecules to amplify the functions of NK cells, thereby improving their therapeutic efficacy (42).

#### 3.3.2 CAR structure

**Figure 3:**
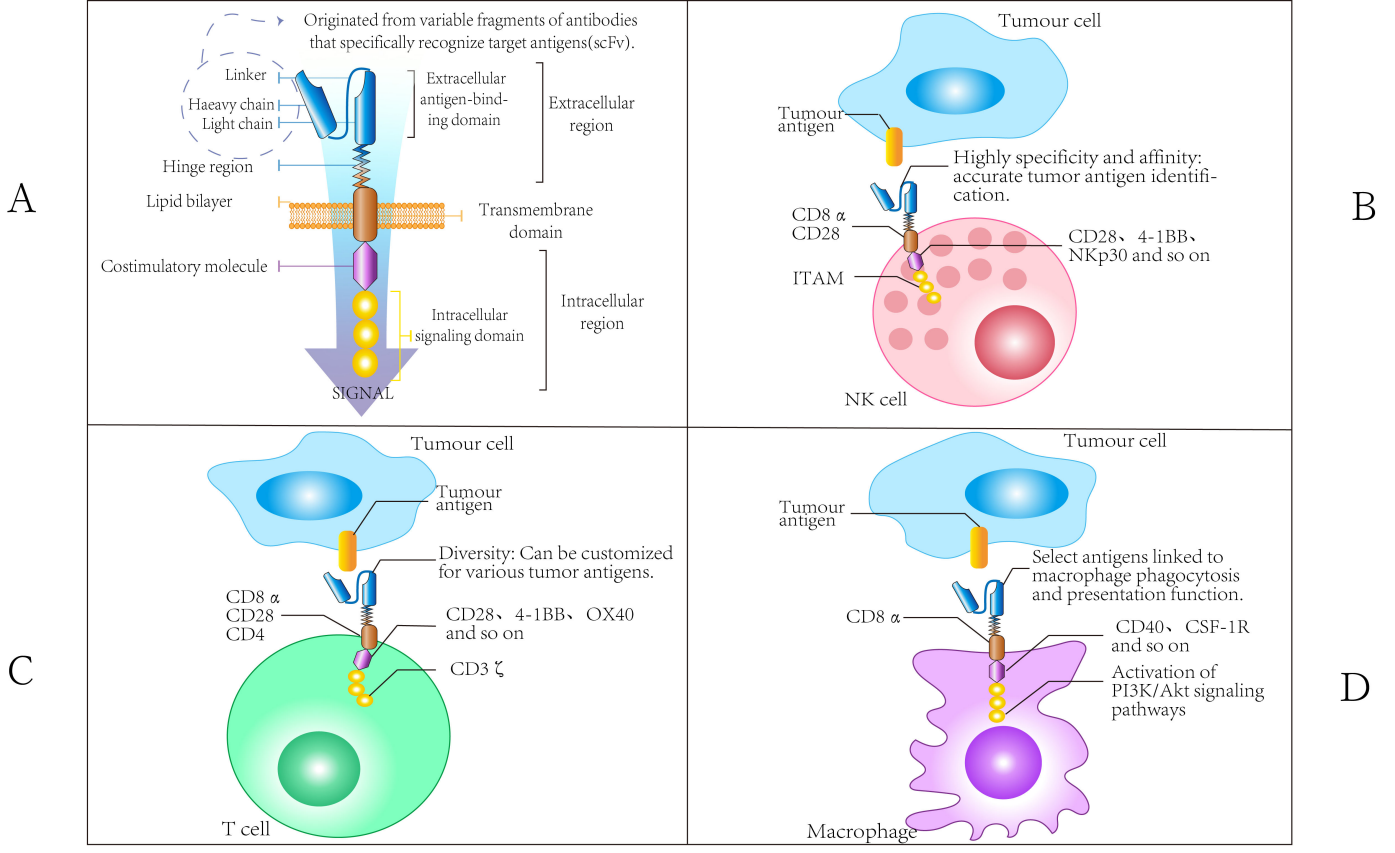
Chimeric Antigen Receptor Domain. **(A)** The CAR domain comprises an extracellular antigen-binding domain (including both heavy and light chains), a hinge region, a transmembrane domain, and an intracellular region, exhibiting high specificity, affinity, and diversity. Additionally, a variety of costimulatory molecules, such as CD28, play crucial roles in interactions between immune cells and can activate associated signaling pathways. **(B)** The CAR molecules present on NK cells consist of scFv that specifically recognize tumor antigens, transmembrane domains (such as CD8α), and intracellular signaling domains (including ITAM). The scFv is characterized by high specificity and diversity, allowing for the accurate recognition of various tumor antigens. The transmembrane domain serves to connect the extracellular environment to the intracellular space, with its component characteristics influencing the localization and function of CAR molecules on the cell membrane. The ITAM structure within the intracellular signaling domain is crucial for signaling processes. By interacting with other molecules, it activates the cytotoxic functions of NK cells, enabling them to identify and eliminate tumor cells. **(C)** The CAR molecules of T cells also incorporate scFv for antigen recognition. The components of the transmembrane domain may differ from those found in NK cells, resulting in distinct molecular compositions that influence their interactions with other cells. When the intracellular signaling domain activates relevant signaling pathways, such as PI3K/Akt, it is linked to the specific functions of T cells. For instance, during the immune response, the activation, proliferation, and differentiation of T cells are intricately regulated by CAR molecular signaling. This process differs from the signaling mechanisms of CAR molecules in NK cells, highlighting the distinct impact on cell function. **(D)** The scFv component of the CAR molecular structure in macrophages facilitates the specific recognition of tumor antigens. The transmembrane domain may contain macrophage-specific molecular elements that influence the stability of CAR molecules and their interactions with other cell surface proteins. The intracellular region, in conjunction with costimulatory molecules such as CD40 and CSF-1R, primarily regulates the phagocytic function of macrophages, enhancing their ability to engulf tumor cells. This regulatory mechanism is notably distinct from the functional modulation of CAR molecules observed in NK cells and T cells.

A chimeric antigen receptor (CAR) typically comprises four structural domains: the extracellular antigen-binding domain, the spacer or hinge region, the transmembrane domain, and the intracellular signaling domain (43). The extracellular antigen-binding domain consists of a single-chain variable fragment (scFv), which is formed by linking the variable heavy chain (VH) and variable light chain (VL) of an antibody through a flexible linker. This domain is crucial for determining the specificity of antigen binding. The spacer region, also referred to as the hinge region, facilitates the exposure of the antigen-binding domain on the surface of CAR-engineered immune cells and serves as an intercellular space between target cells and their antigens. The transmembrane domain anchors the CAR to the cell membrane of immune cells, influencing CAR expression, stability, dimerization, and signal transduction. Finally, the intracellular signaling domain is essential for transmitting signals that activate immune cells to attack target cells (43–45) (**Figure 3**).

#### 3.3.3 Advantages of CAR-NK

Chimeric antigen receptor (CAR) technology initially demonstrated efficacy in T cells and has been employed in the treatment of relapsed/refractory (R/R) B-cell acute lymphocytic leukemia (B-ALL), non-Hodgkin lymphoma (NHL), and multiple myeloma (MM) (46, 47). The application of CAR technology to natural killer (NK) cells holds promise for effectively alleviating the side effects associated with Chimeric Antigen Receptor T-cell (CAR-T) treatment (**Table 1**).

**Table 1:** The difference between CAR-NK, CAR-T and CAR-M.

	CAR-NK	CAR-T	CAR-M
Source	Obtained from multiple sources: PBMCs; NK cell line; UCB; iPSC.	Extract from the patient’s own blood, then perform genetic modification and amplification	Induced differentiation of peripheral blood mononuclear cells
Cellular characteristics	Innate immunity, no need for sensitization, not restricted by MHC	Specific immunity, strong specificity and memory, restricted by MHC	Diverse immune functions include phagocytosis and presentation, polarization being influenced by the microenvironment.
Preparation difficulty	Preparation of the product is relatively simple and can be made into “ready-made” products without patient cell preparation.	Personalized treatment adds complexity and uncertainty to preparation, with a long preparation cycle of several weeks.	Gene editing and transduction have low efficiency and *in vitro* culture can change functions, making precise control of conditions necessary.
Immunogenicity	NK cells maintain low immunogenicity post-genetic modification due to their inherent characteristics.	Non-self CAR proteins expressed from externally introduced genes can lead to GVHD.	The immunogenicity was low, but it was greatly affected by the tumor microenvironment
Duration of existence	Can exist for several days to weeks	Surviving long in the body and exerting anti-tumor effects continuously provide long-term immune protection.	Can exist for several days to weeks
Off-target effects	Adverse reactions are mild and immune response is usually limited even with off-target occurrences.	May cause severe immune reactions, worsen symptoms, and endanger lives.	There is a risk of off-target effects, which are mainly related to antigen recognition specificity and tumor microenvironment.
Adverse reactions	Serious adverse reactions like CRS and neurotoxicity are relatively low.	CRS and neurotoxicity are prone to occur during the process of killing tumor cells	Safer, polarization affected by microenvironment
Clinical application status	Potential application advantages in the treatment of solid tumors.	Hematological tumors treated with significant therapeutic effects achieved.	Preclinical and early clinical trials have potential for solid tumors

First, NK cells can target and eliminate cells that downregulate MHC I molecules, which are not recognized by T cells (48). Second, CAR-NK cells are activated independently of the MHC pathway, thereby circumventing the risk of graft-versus-host disease (GVHD) (49) that can arise from allogeneic CAR-T therapies (50). This characteristic enables CAR-NK cells to address the challenges posed by prolonged culture times commonly associated with autologous cell therapies through the use of NK cell lines and induced pluripotent stem cells (iPSCs) (51, 52). Thirdly, T cell activation results in the production of substantial amounts of inflammatory cytokines, including tumor necrosis factor-alpha (TNF-α), interleukin-1 beta (IL-1β), interleukin-2 (IL-2), and interleukin-6 (IL-6). This cascade of cytokine release can lead to cytokine release syndrome (CRS) and neurotoxicity. In contrast, while NK cell activation also produces pro-inflammatory cytokines such as interferon-gamma (IFN-γ), interleukin-3 (IL-3), and TNF-α, the quantities generated are relatively small, which helps to mitigate toxicity. Additionally, the absence of the critical cytokine IL-6 makes it less likely for CRS to occur (53). Finally, CAR-NK cells employ a variety of mechanisms to eliminate target cells, including activating and inhibitory receptors, as well as CD16-mediated antibody-dependent cell cytotoxicity (ADCC) (54, 55).

#### 3.3.4 Limitations of CAR-NK

The selection of target antigens presents significant challenges. Ideal antigens are those that are tumor-specific, rather than those expressed in normal cells; however, most solid tumors lack traditional tumor-specific antigens, complicating target selection (56). Currently, only EGFR III and tumor neoantigens satisfy these criteria (57). Alternatively, when side effects are manageable-such as with CD19 (a B-cell surface marker) and BCMA (a plasma-cell surface marker), which are also expressed in normal tissues-CAR-T therapy can target these antigens, leading to B-cell deficiency or depletion, which can be mitigated through clinical management, such as immunoglobulin replacement therapy. Furthermore, the use of CAR-NK cells in blood cancers benefits from the characteristics of the blood system, allowing for tolerance of on-target, off-tumor toxicity (OTOT) (58). However, solid organ tumor therapies do not enjoy such advantages (59, 60). In the presence of OTOT, non-renewable or difficult-to-regenerate solid organ cells may experience pronounced side effects (61).Killer-cell immunoglobulin-like receptors (KIRs) can limit the ability of NK cells to attack tumor cells that exhibit high levels of MHC class I (62). Additionally, the immunosuppressive tumor microenvironment (TME) can impair the function of CAR-NK cells (63, 64). Lastly, due to the brief lifespan of CAR-NK cells, repeated infusions are necessary to maintain remission. CAR-NK cells expressing IL-12, IL-15, and IL-18 can extend their survival (65).

### 3.4 NK cell gene editing technology

Five key considerations in cytokine gene editing for enhancing NK cell therapy include: cytokine gene editing, activation of receptor expression, knockout of inhibitory receptors, regulation of immune checkpoints, and anti-apoptotic gene editing.

Cytokine transfection is a technique that utilizes gene editing to introduce or amplify specific cytokines, such as IL-15 and IL-2, to enhance NK cell survival, proliferation, and function, thereby augmenting their anti-tumor effects (64). Upregulating or introducing activating receptors can strengthen activation signals, enhancing NK cell recognition and destruction of tumor cells (65). Gene editing methods, including CRISPR, can be employed to knock out inhibitory receptors, such as NKG2A and PD-1, thereby reducing inhibitory signals and enhancing NK cell activity and anti-tumor potential (66). Additionally, gene editing technology can be utilized to regulate the expression of immune checkpoint molecules, diminishing NK cell inhibition and further amplifying their immune response to tumors (67, 68).

In summary, gene editing technology has opened new avenues for enhancing NK cell function, improving their anti-tumor activity, and developing innovative therapeutic strategies. As research progresses, the clinical translation of engineered NK cells is anticipated to provide significant benefits in the treatment of various cancers and other diseases.

### 3.5 NK cell combination therapy

Research on the integration of NK cells with other immunotherapies, radiotherapy, and chemotherapy is steadily increasing, demonstrating promising clinical application prospects. The combination of NK cells with immune checkpoint inhibitors has been shown to enhance the immune response within the tumor microenvironment and to counteract the mechanisms by which tumor cells evade the immune system (69). The combination of anti-PD-1/PD-L1 antibodies with NK-cell therapy enhances the inhibition of signaling pathways by blocking PD-1/PD-L1 interactions in NK cells. This strategy also amplifies the antibody-dependent cellular cytotoxicity (ADCC) effect of NK cells, thereby significantly improving outcomes in MHC-deficient tumors characterized by low antigenicity or resistance to T cell recognition and lysis. The results were evident, with a doubling of NK cell counts, a substantial increase in cytokine levels such as IL-2, TNF-β, and IFN-γ, and a marked decrease in various tumor markers, including circulating tumor cells (CTC). Patients receiving the combination therapy exhibited a significantly higher overall response rate (36.5% vs. 18.5%) and improved survival outcomes (overall survival: 15.5 months vs. 13.3 months; progression-free survival: 6.5 months vs. 4.3 months; all p< 0.05) compared to those treated with anti-PD-1 antibodies alone (70). Regarding radiotherapy, it can induce tumor cells to release various immunogenic substances, thereby enhancing NK cell activity and increasing tumor cell sensitivity (71, 72). Kim et al. demonstrated that the long-term migration and infiltration of natural killer (NK) cells to the primary tumor site were significantly enhanced following the combination of local tumor radiotherapy (RT) and NK cell therapy. This combination notably inhibited metastasis to the axillary lymph nodes, liver, and lungs (axillary metastases in the RT group, NK group, and RT + NK group were 85%, 20%, and 0%). Furthermore, the long-term survival rate of the RT + NK group was significantly higher than that of either the RT or NK group alone, with an 80-day survival rate of 0% for the RT group, 40% for the NK group, and 80% for the RT + NK group. This finding is supported by Nguyen et al., whose mouse experiments demonstrated that the combination of natural killer (NK) cells and radiotherapy resulted in increased survival rates and reduced tumor metastasis(p<0.0001 and p=0.0287) (73, 74). Additionally, the strategy of combining chemotherapy with NK cells has garnered considerable attention. Certain chemotherapy agents can bolster NK cell effectiveness by directly inducing apoptosis in tumor cells or by downregulating the expression of inhibitory molecules. Chemotherapy not only reduces tumor burden but may also enhance treatment efficacy by creating a more favorable tumor microenvironment for NK cell infiltration and functional expression (74).

In summary, the strategy of combining NK cells with other immunotherapies, radiotherapy, and chemotherapy offers a novel approach to tumor treatment. Future research should further investigate the underlying mechanisms and optimization strategies to achieve enhanced clinical efficacy.

## 4 Application of nanomaterials in NK therapy

While CAR-NK therapy has demonstrated considerable advantages in tumor treatment, it also presents certain limitations, as previously discussed (75). Bioactive nanomaterials, known for their excellent biocompatibility and degradability, serve to complement CAR-NK therapy in the treatment of tumors (76). This synergy not only enhances the efficacy of cancer treatment but also improves its safety profile (77). In the subsequent sections, we will provide a comprehensive discussion on nanoparticle-assisted NK cell therapy from various perspectives, including nanoparticle-assisted immune regulation, the regulation of the tumor microenvironment by nanoparticles, exosome-related nanomaterials, nanoparticle-modified NK cells, and the enhancement of therapeutic efficacy through cell membrane-coated nanomaterials.

### 4.1 Nanoparticles assisted immune regulation

The role of nanoparticles in immune regulation is primarily evident in two key areas. Firstly, they function as carriers for immune modulators, thereby enhancing therapeutic efficacy (78). Due to their extensive delivery range and high efficiency, nanoparticles are capable of encapsulating anticancer drugs, chemokines, and cytokines, facilitating their transport to tumor sites and thereby augmenting the effectiveness of cancer therapies (75, 79). For example, lipid-based nanoparticles loaded with immunomodulatory agents such as TGF-β and IL-2 can enhance immune cell infiltration at tumor locations (80, 81); Alternatively, a dual pH-responsive hydrogel, which contains a tumor acidic neutralizer and neutrophil extracellular trap (NETs) lyase (DNase I), when combined with NK cell infusion, may effectively prevent hepatocellular carcinoma (HCC) recurrence following surgical resection (82). Furthermore, a nanoemulsion system (SSBNMs) designed for the co-delivery of a TGF-β inhibitor and selenocysteine, when co-infused with NK cells, has been shown to improve anti-tumor efficacy. This process relies on the signal transduction of natural killer group 2, member D (NKG2D)/NKG2D ligands (NKG2DLs) and participates in the DNA damage response. Moreover, by inhibiting the TGF-β/TGF-β RI/Smad2/3 signaling pathway, it enhances the expression of NKG2DL on tumor cells, stimulates the surface expression of NKG2D on NK92 cells, and strengthens the immune response (83, 84).

**Figure 4:**
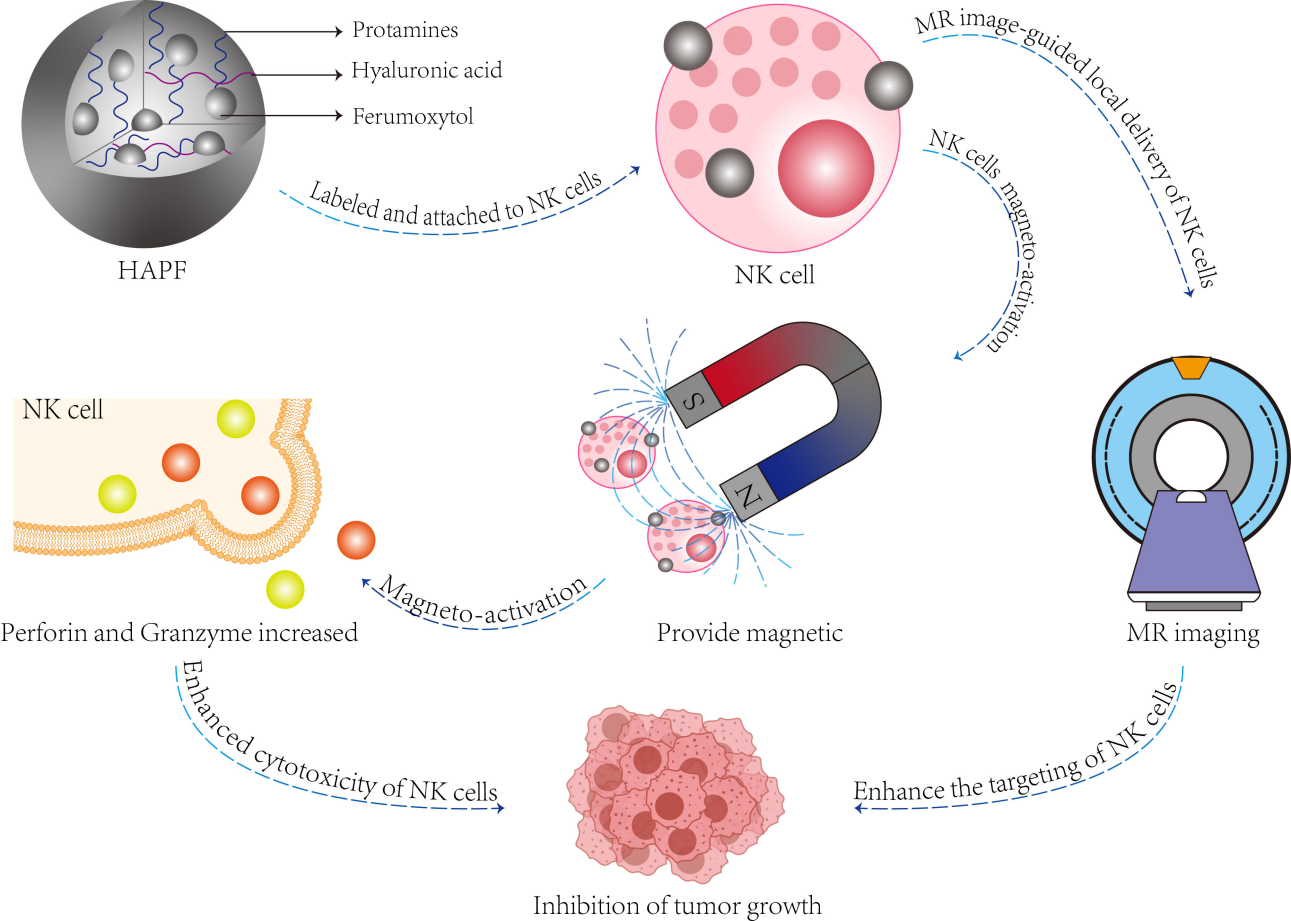
The function and composition of HAPF nanocomplex. The HAPF nanocomplex, composed of hyaluronic acid, protamine, and iron oxide, effectively attaches to natural killer (NK) cells (referred to as HAPF-NK). The application of exogenous magnetic fields facilitates the magnetic activation of NK cells, enhancing the production and secretion of perforin and granzyme within these cells, thereby achieving a cytotoxic effect on target cells. Additionally, magnetically activated HAPF-NK cells enable magnetic resonance (MR) image-guided NK cell therapy for the treatment of solid tumors.

Secondly, nanoparticles significantly enhance the homing of natural killer (NK) cells. The homing process of immune cells is mediated by the release of cytokines and chemokines from tumor cells, which induces inflammation and activates cytotoxic immunity. This dynamic process continuously recruits immune cells into the tumor microenvironment, ultimately leading to the destruction of cancer cells (82). The incorporation of magnetic nanomaterials onto the surface of NK cells facilitates the targeted attraction of these cells to tumor sites, guided by an external magnetic field (85, 86). Magnetic nanomaterials can be categorized into three distinct types (87): firstly, magnetic pure metals (Fe, Co, Ni), which offer the advantage of easy synthesis but are prone to oxidation and combustion in air; secondly, magnetic metal oxides (Fe2O3, Fe3O4) or ferrites (MeFe2O4, me=Fe, Co, Zn) possess excellent magnetic properties and low toxicity (88, 89); and third, multicomponent magnetic nanoparticles, such as core/shell magnetic nanoparticles (MNPs) or magnetic nanoclusters, which overcome the limitations of single-component materials and introduce novel functionalities (90). In a separate study, a therapeutic ‘biohybrid’ (NK: IONP) was developed by conjugating iron oxide nanoparticles (IONPs) to umbilical cord blood-derived NK cells using advanced biological coupling technology, thereby imparting enhanced magnetic guidance capabilities to NK cells (91). Additionally, magnetic nanoparticles not only do not impair NK cell function but also exhibit an activating effect on these cells. A magnetic nanocomposite: hyaluronic acid-protamine-ferumoxytol(HAPF) composed of hyaluronic acid (HA), protamine (P), and ferumoxytol (F) has been formulated, which effectively activates NK cells under the application of an external magnetic field, promotes the secretion of perforin and granzyme, and enhances the efficacy of tumor cell killing (92) (**Figure 4**).

### 4.2 Nanoparticles regulate tumor microenvironment

As previously mentioned, the accumulation of lactate and adenosine in the tumor microenvironment, along with hypoxia, can create an immunosuppressive milieu that impairs the cytotoxic function of NK cells (93). Therefore, reversing immune suppression within the tumor microenvironment is crucial for the efficacy of NK cell therapy (94). In comparison to conventional treatment modalities such as radiotherapy, chemotherapy, and immunotherapy, nanomaterials present distinct advantages. Research has shown that poly(lactic acid glycolic acid) copolymer (PLGA) - MnO2 nanoparticles alleviate hypoxia and reduce the levels of potent immunosuppressive metabolites like lactate and adenosine upon penetrating cancer spheroids (95); Moreover, vesicular cationic lipid-assisted nanoparticles (CLAN) inhibit the function of the CD47 molecule (96), effectively activate dendritic cells (97), decrease lactate secretion, normalize tumor acidity, enhance immune cell infiltration, and restore the anti-tumor response of T cells and NK cells (98). Additionally, drugs delivered via nanomaterials can also hinder the activation of tumor fibroblasts, reduce extracellular matrix accumulation, and improve the tumor microenvironment (99).

Furthermore, the enhancement of NK cell infiltration at the tumor site can be facilitated by increased release of cytokines and chemokines (100). For instance, These nanowire antibody substrates were able to locate endogenous IL-2 in the skin, increase endogenous IL-2 levels, and activate targeted natural killer cells, enabling tissue-specific and cell-specific immune activation (101).

### 4.3 Exosome related nanomaterials

Exosomes are spherical nano-vesicles secreted by cells, composed of lipid membranes (102). These subcellular vesicles are encapsulated by a phospholipid bilayer membrane and contain a variety of carriers, including miRNA, mRNA, DNA, and proteins, which play significant roles in the physiological and pathological processes of the body (103). Exosomes exhibit certain anti-tumor activities (104). For instance, NK cell-derived exosomes (NDEs) can clear tumor cells and stimulate adaptive immunity by secreting chemokines and pro-inflammatory cytokines (105, 106); DC cell-derived exosomes (DEXs) can serve as tumor vaccines that kill tumors through T cell-dependent and MHC-restrictive mechanisms (107, 108). Additionally, they can capture tumor-associated antigens (TAAs) and promote immune cell-dependent tumor rejection (109). Intriguingly, tumor cell-derived exosomes (TDES) not only exert immunosuppressive effects and induce M2 polarization of macrophages (110) but also participate in cytotoxic immune responses via antigen presentation or direct activation of NK cells, leading to the elimination of tumor cells (111).

Nucleic acids carried in exosomes can also enhance the efficacy of tumor therapy (112, 113). RNA derivatives such as siRNA, miRNA, and shRNA can silence specific genes, thereby enhancing NK cell activity (114). This approach falls under RNA interference (RNAi)-mediated immunotherapy (115). For example, the *in vivo* and intracellular transportation of siRNA requires superior carriers to overcome its short half-life and prevent degradation (116). With advancements in technology, lonizable cationic lipid nanoparticles can effectively combine with negatively charged siRNA, resulting in siRNA-lipid nanoparticle (siRNA-LNP) complexes that exhibit remarkable stability, making them the preferred carriers at present (117). Furthermore, NK cell-derived exosomes can be combined with miRNA-loaded biomimetic core-shell nanoparticles (NN) for targeted therapy, which demonstrates a dual inhibitory effect on tumor growth (118).

Exosomes face limitations in large-scale production and are readily captured by the liver *in vivo*, which significantly diminishes their delivery efficiency (119). Consequently, researchers are exploring alternative approaches, such as Plant-Derived Exosomes-Like Nanoparticles (PELNs), which have shown considerable promise in immune regulation (120), PELNs offer unique advantages, including evasion from immune detection, enhanced bioavailability, and reduced side effects (117). Currently, this technology has demonstrated substantial efficacy in the treatment of inflammatory diseases; however, research in this area remains insufficient, and many underlying mechanisms are not yet fully understood. This represents a promising avenue for future research on nanoparticles in tumor treatment (121).

### 4.4 Nanoparticle modified NK cells

Nanoparticles can significantly enhance the efficacy of immunotherapy by modifying natural killer (NK) cells. These nanomaterials are capable of delivering chimeric antigen receptor genes to patient-derived NK cells, thereby potentiating the advancement of CAR-NK cell therapy (122). For instance, the multifunctional nanoparticles (MF-NPs) effectively deliver genetic material to immune cells, induce the expression of targeted chimeric antigen receptors (EGFR-CARs) on the surface of NK cells, and improve the anticancer cytotoxicity of the cells *in vitro* and *in vivo* (123). Furthermore, nanoparticles serve as a crucial tool for engineering the surface of NK cells (124). Recently, there has been an increasing focus in this area: the core-shell membrane-fusogenic liposome (MFL) can enhance the levels of NK-activated free oligosaccharides while suppressing the immunosuppressive glycans on the surface of tumor cells through membrane fusion (125). Nanobody 7D12, characterized by its high affinity for antigens, low immunogenicity, and improved penetration into tumors, is conjugated with NK cells via orthogonal click chemistry. The resulting 7D12-NK92MI constructs can specifically target and eliminate solid tumor cells that overexpress EGFR (126). In conclusion, the potential of nanomaterial-mediated NK cell modification represents a promising research trajectory in NK cell therapy.

### 4.5 Cell membrane coated nanomaterials enhance therapeutic efficacy

Cell membrane-coated nanomaterials (CNPs) consist of a synthetic nanoparticle core that is camouflaged by a natural cell membrane. These materials are adept at functioning within complex biological environments and can significantly enhance the efficacy of nanomaterials (127). Nanomaterials enveloped in natural cell membranes exhibit the advantageous properties of both cell membranes and nanomaterials, including improved biocompatibility and targeted cellular delivery (128). Furthermore, the functions derived from membranes of different cellular origins possess unique characteristics. For example, macrophage- or neutrophil-coated nanomaterials can interact with tumor tissue to inhibit cancer progression and metastasis (129). In contrast, nanoparticles modified with red blood cell membranes have a longer half-life and are less likely to be recognized and eliminated by the immune system (130). Additionally, NK cell membrane-coated nanomaterials can greatly enhance targeting capabilities (131),while biomimetic NK cell nanomaterials (DMLN) can overcome multidrug resistance (132). Depending on specific therapeutic needs, various cell membrane modifications can be selected to augment the effects of NK cell therapy, and hybrid cell membrane modifications can also be employed to achieve a range of functions (130).

## 5 Conclusion and perspectives

NK cell therapy has demonstrated potential in tumor treatment, yet its clinical translation remains challenging. While CAR-NK technology avoids the side effects of CAR-T (e.g., GVHD and CRS), critical issues such as target antigen selection, off-target toxicity in solid tumors, immunosuppressive microenvironments, and short cell lifespan urgently require resolution. Gene editing technologies (e.g., CRISPR) and combination therapies (immune checkpoint inhibitors, chemoradiotherapy) offer new directions for optimizing NK cell function.

The application of nanomaterials has significantly enhanced the efficacy of NK therapy. Nanoparticles can improve treatment by delivering immunomodulators (e.g., TGF-β inhibitors), enhancing NK cell homing (via magnetic nanomaterials), and modulating the tumor microenvironment (e.g., neutralizing lactate/adenosine). Exosome-related nanomaterials exhibit anti-tumor activity, but breakthroughs are still needed in their large-scale production and targeted delivery. Plant-derived nanomaterials (e.g., PELNs), with their low immunogenicity and high biocompatibility, may become a future priority. Additionally, nanomaterial-modified NK cells (e.g., membrane-fused liposomes) and biomimetic membrane coatings (e.g., macrophage membranes) further expand functional diversity (**Table 2**).

**Table 2:** The Functions and Applications of Nanomaterials.

Nanomaterials		Functions and Applications	References
Nanoparticles carrying immune modulators		Enhance cancer therapy efficacy by encapsulating drugs or chemokines and cytokines to transport them to the tumor site.	(78)
Lipid nanoparticles		Loaded with immune modulators such as TGF-β and IL-2 and Promote immune cell infiltration at tumor sites.	(80, 81)
Dual pH-responsive hydrogel		Featuring a potent acidic tumor neutralizer and NET lyase, the treatment is designed to reduce the likelihood of Hepatocellular Carcinoma recurring post-resection, utilizing the additional support of NK cell infusion.	(82)
Nanoemulsion system (SSBNMs)		Used for synergistic co-delivery of TGF-β inhibitors and selenocysteine, co-infused with NK cells to amplify anti-tumor effects.	(83, 84)
Magnetic pure metals (Fe, Co, Ni)		In combination with magnetic field guidance, this nanomaterial can be employed to alter NK cells in order to attract them to tumor sites for targeted therapy.	(87)
Magnetic metal oxides (Fe2O3, Fe3O4) or ferrites (MeFe2O4, me = Fe, Co, Zn)		Exhibit superior magnetic properties and low toxicity, whilst also being able to partner with NK cells and magnetic field guidance for precise cancer treatment. Additionally, they have the capability to enhance NK cell functionality.	(88, 89)
Multicomponent magnetic nanoparticles (such as core/shell MNPs or magnetic nanoclusters)		Enhanced the performance of single-component materials by compensating for their limitations and providing novel functionalities.	(90)
NK: INOP		Provides additional magnetic guidance capabilities.	(91)
Nanocomposite (HAPF) consisting of hyaluronic acid (HA), protamine (P), and ferumoxytol(F)		Effectively activate NK cells, promote the secretion of perforin and granulozyme, and enhance the killing effect of tumor cells.	(92)
Poly(lactic acid glycolic acid) copolymer (PLGA) -MnO2 nanoparticles		To lessen the levels of hypoxia and potent immunosuppressive metabolites such as lactate and adenosine within the tumor microenvironment.	(95)
Vesicular cationic lipid-assisted nanoparticles (CLAN)		Decreased the function of the CD47 molecule, thereby stimulating the activation of dendritic cells, reducing lactate production, normalizing tumor acidity, promoting the infiltration of immune cells, and ultimately restoring the anti-tumor response of T cells and NK cells.	(96, 97)
Nanowires		Conjugate with an anti-IL-2 antibody, augment endogenous IL-2 concentrations, and leverage these alterations to induce the recruitment and activation of natural killer cells.	(101)
Plant-Derived Exosomes-Like Nanoparticles (PELNs)		Play an important role in immune regulation and are currently employed in the treatment of inflammatory diseases.	(120)
multifunctional nanoparticles (MF-NPs)		EGFR expression is induced on the surface of NK cells targeting chimeric antigen receptors (EGFR-CARS)	(123)
The core-shell membrane-fusogenic liposome (MFL)		Membrane fusion increased the level of free oligosaccharides activated by NK cells and inhibited immunosuppressive glycans on the surface of tumor cells.	(125)
The nanobody is coupled with NK cells to form 7D12-NK92MI		Specifically target and eliminate solid tumor cells with EGFR overexpression	(126)
Ionizable cationic lipid nanoparticles		Efficiently combines with negatively charged siRNA to generate a product named siRNA-LNP. Furthermore, due to its exceptional stability, the siRNA-LNP has become a preeminent carrier for RNAi-mediated immunotherapy.	(117)
Cell membrane coated nanomaterial (CNP)	CNP with macrophage or neutrophil cell membrane coating	Interact with tumor tissue to inhibit cancer progression and metastasis.	(129)
	CNP with red blood cell membrane coating	Have a prolonged half-life and are less likely to be identified and eliminated by the immune system, thereby increasing their effectiveness.	(130)
	CNP with NK cell membrane coating	Provide improved targeting capabilities and have the potential to overcome multidrug resistance.	(131, 132)

Future research should focus on: 1.Optimizing CAR-NK targeting strategies to reduce off-target toxicity; 2.Elucidating nanomaterial mechanisms, developing low-toxicity and high-efficiency materials, and addressing production/delivery challenges; 3.Exploring synergistic effects of combining nanomaterials with NK therapy to identify more effective regimens; 4.Advancing the clinical translation of plant-derived nanomaterials and engineered exosomes. These efforts will accelerate breakthroughs in NK therapy for solid tumors, providing better options for cancer patients.
